# Adults with diabetes residing in “food swamps” have higher hospitalization rates

**DOI:** 10.1111/1475-6773.13102

**Published:** 2019-01-06

**Authors:** Aryn Z. Phillips, Hector P. Rodriguez

**Affiliations:** ^1^ University of California Berkeley Berkeley California; ^2^ Center for Healthcare Organizational and Innovation Research University of California Berkeley California

**Keywords:** diabetes, disparities, food swamps, hospitalizations, rurality

## Abstract

**Objective:**

To examine the relationship between food swamps and hospitalization rates among adults with diabetes.

**Data Sources:**

Blue Cross Blue Shield Association Community Health Management Hub^®^ 2014, AHRQ Health Care Cost and Utilization Project state inpatient databases 2014, and HHS Area Health Resources File 2010‐2014.

**Study Design:**

Cross‐sectional analysis of 784 counties across 15 states. Food swamps were measured using a ratio of fast food outlets to grocers. Multivariate linear regression estimated the association of food swamp severity and hospitalization rates. Population‐weighted models were controlled for comorbidities; Medicaid; emergency room utilization; percentage of population that is female, Black, Hispanic, and over age 65; and state fixed effects. Analyses were stratified by rural‐urban category.

**Principal Findings:**

Adults with diabetes residing in more severe food swamps had higher hospitalization rates. In adjusted analyses, a one unit higher food swamp score was significantly associated with 49.79 (95 percent confidence interval (CI) = 19.28, 80.29) additional all‐cause hospitalizations and 19.12 (95 percent CI = 11.09, 27.15) additional ambulatory care‐sensitive hospitalizations per 1000 adults with diabetes. The food swamp/all‐cause hospitalization rate relationship was stronger in rural counties than urban counties.

**Conclusions:**

Food swamps are significantly associated with higher hospitalization rates among adults with diabetes. Improving the local food environment may help reduce this disparity.

## 1 INTRODUCTION

Health care providers and systems are increasingly being incentivized to improve the management of chronic conditions while simultaneously reducing costs of care. Adults with diabetes have garnered a significant amount of attention because diabetes and its complications are often preventable yet are very costly to treat once they occur. For instance, the Centers for Medicare and Medicaid Services (CMS) estimates that in 2016 it spent an additional $42 billion on beneficiaries with diabetes than it would have had on these beneficiaries not had diabetes. The largest proportion of this expenditure is on hospital services; $3100 is spent per beneficiary on hospital services vs $1500 for prescription drugs and $2700 for physician/clinical services.^1^ As a result, CMS has developed the Medicare Diabetes Prevention Program Model as one of its Innovation Models, its experimental payment and service delivery models aimed at achieving better care at lower costs.^1^

Management of diabetes and its complications is highly dependent on dietary intake. The quantity and quality of foods consumed affects one('s blood glucose levels, and untreated hyperglycemia in diabetics can lead over time to cardiovascular disease, kidney disease, and infection from damaged blood vessels as well as other emergency complications such as diabetic ketoacidosis and hyperglycemic hyperosmolar syndrome.^2^ As such, individuals with diabetes are encouraged to limit their intake of processed carbohydrates, saturated and trans‐fatty acids, cholesterol, and sodium.^3^

Dietary intake, however, is impacted by the nature of the local food environment. Distance to and density of neighborhood grocery stores, fast food outlets, and convenience stores have been found to be associated with fruit and vegetable consumption and other dietary quality measures as well as with obesity.^4^, ^5^, ^6^ Research in this area emphasizes that the availability of both healthy and unhealthy foods can be influential in predicting dietary outcomes, leading some recent studies to focus on the number of unhealthy outlets relative to the number of healthy outlets as a measure of the food environment rather than absolute measures. The term “food swamps” was coined in 2009 by Rose et al^7^ to describe those areas with high relative measures, defining them as places in which large numbers of unhealthy energy‐dense food offerings inundate or “swamp out” the relatively few existing healthy food offerings. Studies using relative food environment measures as predictors have found that food swamps have modest but significant associations with obesity^6^, ^8^, ^9^, ^10^ and that these associations may perhaps be stronger than those of “food deserts,” which are areas in which residents must travel long distances to reach grocery stores.^11^

If food swamps are associated with obesity through dietary intake, it is likely that, in addition, adults with diabetes residing in food swamps are more vulnerable and prone to diabetic exacerbations and complications caused by poor dietary intake. If so, this vulnerability may be placing diabetics living in these areas at a distinct disadvantage that exists entirely separate from the health care system and creating a disparity in health outcomes and service utilization.

This analysis assesses the degree to which counties in which large relative numbers of outlets selling energy‐dense foods overwhelm healthy food options have significantly higher hospitalization rates among adults with diabetes, controlling for other area health system‐related and sociodemographic characteristics. Further, we examine whether or not the food swamp‐hospitalization rate relationship varies in urban and rural counties. Urban and rural areas differ markedly in their transportation resources and the types of retail outlets that choose to locate within them.^12^, ^13^, ^14^ It is, therefore, possible that the association is stronger in rural areas, where supermarkets and robust public transportation systems that facilitate access are lacking.

Some previous work has studied the association between food environment and diabetes prevalence^8^, ^15^, ^16^ and incidence,^17^, ^18^ but, to our knowledge, the relationship between food environment and hospital utilization among adults with diabetes has not been assessed. This study adds to the limited research using relative food environment measures in the U.S. context, particularly across multiple geographic areas.

## 2 METHODS

### 2.1 Data

Data for this analysis were accumulated from a variety of public and proprietary sources for the year 2014. Food environment data were obtained from the Blue Cross and Blue Shield Association (BCBSA) Community Health Management Hub (CHM Hub^®^). The CHM Hub^®^ is a proprietary database created and maintained by BCBSA that contains information on patient health outcomes as well as physical and socioeconomic neighborhood characteristics for all zip codes in the United States. Data are amassed from Blue Cross and Blue Shield health plan claims as well as the American Community Survey, the North American Industry Classification System, the USDA Economic Research Service, the Bureau of Transportation Statistics, and Nielsen Homescan. Additional data on the local food environment used in sensitivity analysis are from the USDA Food Environment Atlas. Data on adult diabetic hospitalization rates came from the Agency for Healthcare Research & Quality (AHRQ) Health Care Cost and Utilization Project (HCUP) state inpatient databases and the Centers for Disease Control and Prevention (CDC) Behavioral Risk Factor Surveillance Survey (BRFSS). The HCUP state inpatient databases (SID) contain discharge records for community hospital inpatient stays, regardless of payer, within participating states,^19^ and the CDC uses BRFSS to estimate yearly county‐level diabetes prevalence rates.^20^ Data on relevant sociodemographic factors, including rural‐urban categorization, were obtained from the Health and Human Services (HHS) Area Health Resources Files (AHRF), a county‐level database of health care‐related and other contextual information that is compiled from over 50 different sources.^21^ Data were linked using county Federal Information Processing System (FIPS) codes.

The analytic sample includes 784 counties in 15 states: AZ, AR, CO, FL, GA, IA, MA, MI, NJ, NM, NY, OR, RI, VT, and WA, which were included based on HCUP SID availability and represent a variety of states with regard to region, size, population demographics, etc. With the exclusion of two outliers, all counties from each state were included.

### 2.2 Measures

#### 2.2.1 Outcome

This analysis examines two measures of hospitalization rates among adults with diabetes: rates of all‐cause hospitalizations and rates of hospitalizations for ambulatory care‐sensitive conditions (ACSC).

All‐cause hospitalizations refer to inpatient hospitalizations among adults with diabetes over the age of 20 for any reason over the course of the year. Rates were calculated by summing all admissions with any‐listed diagnosis of Clinical Classification Software code 49 (“diabetes mellitus without complication”) or 50 (“diabetes mellitus with complications”). Common principal diagnoses for all‐cause admissions include septicemia, pneumonia, kidney failure, subendocardial infarction, and osteoarthrosis of the leg. Sums were divided by each county's diabetes prevalence rate as estimated by the CDC BRFSS. All‐cause hospitalizations were analyzed to assess potential spillover effects of the local food environments on health care utilization; poor glycemic control among adults with diabetes can result in complications that may initially seem unrelated to diabetes, such as those listed above, and these diagnoses may not be marked specifically as diabetes complications in inpatient records.^22^

The ACSC hospitalization rate considers only admissions with a principal diagnosis that meets the AHRQ Prevention Quality Overall ACSC Composite specifications. These diagnoses include diabetes with short‐term complications, diabetes with long‐term complications, uncontrolled diabetes without complications, diabetes with lower‐extremity amputation, chronic obstructive pulmonary disease, asthma, hypertension, heart failure, dehydration, bacterial pneumonia, or urinary tract infection. This measure was included because these diagnoses are preventable through access to high‐quality ambulatory care services^23^, ^24^ and exacerbated diabetes often manifests itself in such conditions.^25^

Rates are presented as the number of hospitalizations per every 1000 adults with diabetes.

#### 2.2.2 Main independent variables

Food swamp severity is measured on a continuous scale. Food swamp scores represent the ratio of the number of fast food outlets to the number of grocers in a county, adjusted for population density and average disposable income and standardized so that values fall between zero and ten. Estimates were calculated at the zip code level by BCBSA. Zip codes were allocated to their respective counties by assigning each to the county in which the majority of its population resides. Food swamp scores were then estimated at the county level by weighted averaging based on the proportion of the county population that each zip code contributes, according to the most recent U.S. Census.

#### 2.2.3 Control variables

Analyses also controlled for several health system‐related and sociodemographic variables that are often associated with hospitalizations among adults with diabetes.^26^, ^27^ Health systems variables include the average number of comorbidities per hospitalized diabetic patient in the county, the percentage of the diabetic population enrolled in Medicaid, and the percentage of the hospitalizations that were admitted to the hospital via the emergency department. Sociodemographic variables include the percentage of the county population that is female, non‐Hispanic black, Hispanic, and over the age of 65.

Rural‐urban categorization was determined according to the methodology used by the U.S. Census Bureau, which places counties into one of three categories based on the percentage of the population that is considered rural as of the 2010 census. Counties are classified as “completely rural” if 100 percent of the population is rural, “mostly rural” if 50‐99.9 percent of the population is rural, and “mostly urban” if less than 50 percent of the population is rural.^28^

### 2.3 Statistical analyses

Multivariate linear regression was used to estimate the association between food swamp severity and hospitalization rates (all‐cause and ACSC) among adults with diabetes at the county level, controlling for health system‐related and sociodemographic covariates. The models included state fixed effects to account for clustering within states and were weighted according to county population so that larger counties contributed more to estimates than smaller counties. The models follow the following specifications:

$$\begin{array}{cc} {\text{All‐cause hospitalization rate}}_{\mathrm{ij}}= & \beta_{1}{\left(\text{food swamp score}\right)}_{\mathrm{ij}}\\ & +\beta_{2}{\left(\text{comorbidity burden}\right)}_{\mathrm{ij}}+\beta_{3}{\left(\%\;\text{Medicaid}\right)}_{\mathrm{ij}}\\ & +\beta_{4}{\left(\text{ED utilization}\right)}_{\mathrm{ij}}+\beta_{5}{\left(\%\;\text{female}\right)}_{\mathrm{ij}}\\ & +\beta_{6}{\left(\%\;\text{black}\right)}_{\mathrm{ij}}+\beta_{7}{\left(\%\;\text{Hispanic}\right)}_{\mathrm{ij}}\\ & +\beta_{8}{\left(\%\;\text{over age}\;65\right)}_{\mathrm{ij}}+\alpha_{j}+\epsilon_{\mathrm{ij}} \end{array}$$

$$\begin{array}{cc} {\text{ACSC hospitalization rate}}_{\mathrm{ij}}= & \beta_{1}{\left(\text{food swamp score}\right)}_{\mathrm{ij}}+\beta_{2}{\left(\text{comorbidity burden}\right)}_{\mathrm{ij}}\\ & +\beta_{3}{\left(\%\;\text{Medicaid}\right)}_{\mathrm{ij}}+\beta_{4}{\left(\text{ED utilization}\right)}_{\mathrm{ij}}\\ & +\beta_{5}{\left(\%\;\text{female}\right)}_{\mathrm{ij}}+\beta_{6}{\left(\%\;\text{black}\right)}_{\mathrm{ij}}\\ & +\beta_{7}{\left(\%\;\text{Hispanic}\right)}_{\mathrm{ij}}+\beta_{8}{\left(\%\;\text{over age}\;65\right)}_{\mathrm{ij}}+\alpha_{j}+\epsilon_{\mathrm{ij}} \end{array}$$

where the hospitalization rate among adults with diabetes for a county (*i*) within a state (*j*) is a function of the food swamp score in county (*i*) in state (*j*), a number of health system‐related and sociodemographic covariates in county (*i*) in state (*j*), an intercept *α*_*j*_ for each state (*j*), and a county‐specific error term *ϵ*. The health system‐related covariates include comorbidity burden, Medicaid enrollment, and emergency department utilization, and the sociodemographic covariates include the proportions of the county population that are female, non‐Hispanic black, Hispanic, and over age 65. Each of these additional covariates had variance inflation factors under 2.0, indicating that collinearity was not an issue for the final models. Income‐related control variables were excluded, as the food swamp variable was already adjusted for average disposable income.

Both models were then stratified by rural‐urban category.

To account for potential selection effects, we estimated the final regression models using inverse propensity treatment weights. To do so, we first created a dichotomous variable classifying a county as a food swamp if its food swamp score was above the median and not a food swamp if it was below the median. Propensity scores were estimated using a number of covariates, including the average comorbidity burden among the diabetic population and the percentage of the population that was non‐Hispanic black, between the ages of 20 and 45, over age 65, had a college degree, on Medicaid, living in urban areas, and living in poverty, all of which either have been linked to poor food environments and hospitalization rates in the literature or varied substantially by food swamp status in this data.

### 2.4 Sensitivity analyses

We conducted a series of sensitivity tests to assess the robustness of the main regression model results. First, as a falsification test,^29^ sometimes called a negative control,^30^ the final model was tested on a clinical outcome that should theoretically be unrelated to diet quality. The outcome chosen was the county rate of hospitalizations with a principal diagnosis of a mood disorder (including bipolar disorder, manic affective disorder, major depressive disorder, and other unspecified mood disorders) among all adults.

Second, to build confidence in the concurrent validity of our food swamp measure, the models were estimated using the Retail Food Environment Index (RFEI) as the predictor. The RFEI consists of the ratio of fast food outlets and convenience stores to grocery stores and supermarkets in an area and has been employed in previous food swamp analyses.^8^, ^9^, ^11^ We constructed RFEI estimates using data from the USDA Food Environment Atlas, divided the ratios by county population estimates, and standardized the variable to achieve greater comparability to the food swamp score. These models were also additionally controlled for median income, as the food swamp score is adjusted for income.

Third, to control for socioeconomic status and area deprivation beyond the incorporated adjustment for median disposable income, we included a measure of the percentage of households in a county that are vacant. Many other measures of deprivation were strongly correlated with the food swamp score and would create collinearity among variables when included the model. The vacant homes variable, however, was only moderately correlated and inclusion resulted in variance inflation factors that all remained below 2.0, so it was added to the model to more strongly control for county socioeconomic status.

Fourth, the use of a ratio as a dependent variable may result in spurious correlation between the dependent variable and the predictor if the predictor is correlated with the ratio's denominator but not with the numerator, conditional on the denominator.^31^ To rule out such correlation, we decomposed the hospitalization rate variable and ran the linear models regressing the number of all‐cause and ACSC hospitalizations (log transformed) on the previously included predictors and the number of adults with diabetes in the county (log transformed).

Finally, to improve causal inference with the cross‐sectional data, an instrumental variable approach was attempted. All instruments, including highway exits, which has been used in previous studies of fast food access,^11^, ^32^ were unsuccessful. While this instrument works well when obesity is the outcome, it did not satisfy the exclusion restriction in this analysis, as highway access is related to transportation which is related to health services access.

## 3 RESULTS

### 3.1 Descriptive statistics

The mean all‐cause hospitalization rate across all counties in the sample was 304.53 hospitalizations per 1000 diabetic adults, but the rate ranged widely from 20.19 hospitalizations per 1000 diabetic adults to 644.25 hospitalizations per 1000 diabetic adults (SD = 86.44). ACSC‐specific hospitalizations were less frequent; the mean rate was 60.72 hospitalizations per 1000 diabetic adults and ranged from 0 hospitalizations to 164.69 hospitalizations per 1000 diabetic adults (SD = 23.53). The mean county food swamp score was 2.77, with a minimum score of 1.69 and a maximum score of 4.35 (SD = 0.37). Descriptive statistics for the contextual variables are presented in Table 1.

**Table 1:** Characteristics of Counties by Rural and Urban Classification

Variable	All counties (n = 784) Mean (SE)	Completely rural (n = 121) Mean (SE)	Mostly rural (n = 309) Mean (SE)	Mostly urban (n = 354) Mean (SE)
*Health system variables*				
Average number of comorbidities per hospitalized adult diabetic	3.94 (0.01)	3.83^a^ (0.04)	3.95 (0.02)	3.98 (0.19)
Percentage of hospitalized adult diabetics on Medicaid	17.21 (0.35)	16.12^a^ (0.88)	16.48^a^ (0.54)	18.23 (0.54)
Percentage of diabetic hospitalizations admitted through ER	39.22 (1.07)	31.22^a^ (2.41)	38.26 (1.54)	42.79 (1.75)
*Sociodemographic variables*				
Percentage of population female	49.87 (0.09)	48.89^a^ (0.33)	49.69^a^ (0.13)	50.37 (0.11)
Percentage of population Non‐Hispanic Black	10.20 (0.53)	8.47 (1.43)	10.87 (0.89)	10.19 (0.71)
Percentage of population Hispanic	9.70 (0.46)	7.68^a^ (1.24)	5.44^a^ (0.41)	14.11 (0.80)
Percentage of population over age 65	18.00 (0.18)	21.32^a^ (0.46)	18.57^a^ (0.22)	16.36 (0.28)

a Denotes *P* < 0.05 significance in test of means compared to mostly urban counties.

*Source*. AHRQ Health Care Cost and Utilization Project (HCUP) state inpatient database (SID) 2014, HHS Area Health Resources File (AHRF) 2010‐2014.

As hypothesized, hospitalization rates and the degree to which counties were food swamps differed by rural‐urban categorization. Hospitalization rates were lower in rural counties. The mean all‐cause rate was only 284.19 hospitalizations per 1000 diabetic adults in completely rural counties compared to 302.08 hospitalizations in mostly rural counties and 313.61 hospitalizations in mostly urban counties. ACSC hospitalization rates were similar in mostly rural and mostly urban counties (63.04 and 61.20 per 1000 diabetic adults, respectively) but substantially lower in completely rural counties (53.33 per 1000 diabetic adults). In contrast, rural counties tended to have more severe food swamps. The mean food swamp score was 3.17 for completely rural counties, 2.87 for mostly rural counties, and 2.56 for mostly urban counties. Descriptive statistics for the control variables by rural‐urban category are shown in Table 1, but, in general, urban areas tended to have patients with more comorbidities, more patients enrolled in Medicaid, and more patients admitted through the emergency department. They also tended to have populations that were more heavily female, black, Hispanic, and younger than rural areas.

### 3.2 Multivariate regression

Results of the multivariate linear regression are displayed in Table 2. Both all‐cause hospitalization rates and ACSC‐specific hospitalization rates among adults with diabetes are significantly higher in counties with poorer quality food environments. More specifically, a one unit higher food swamp score is associated with, on average, an estimated additional 49.79 all‐cause hospitalizations per 1000 diabetic adults (*P* = 0.001, 95 percent confidence interval (CI) = 19.28, 80.29) and an estimated additional 19.12 ACSC hospitalizations per 1000 diabetic adults (*P* < 0.001, 95 percent CI = 11.09, 27.15), controlling for comorbidity burden, Medicaid prevalence, emergency room utilization, and the percentage of the population that is female, black, Hispanic, and over age 65, as well as for state fixed effects. As an alternative interpretation for context, a one standard deviation higher food swamp score is associated with an average estimated additional 18.67 all‐cause hospitalizations per 1000 diabetic adults (*P* = 0.001, 95 percent CI = 7.23, 30.11) and an estimated additional 7.17 ACSC hospitalizations per 1000 diabetic adults (*P* < 0.001, 95 percent CI = 4.16, 10.18), adjusting for covariates.

**Table 2:** The Association of Food Swamps on Hospitalization Rates among Diabetic Adults (N = 784)

* *	All‐cause hospitalization rate	ACSC hospitalization rate
Food swamp score	49.79** (15.54)	19.12*** (4.09)
Comorbidities per patient	94.64*** (16.17)	13.45** (4.22)
Percentage of patients on Medicaid	−1.16 (0.77)	−0.28 (0.25)
Percentage of hospitalizations admitted in ER	−0.14 (0.34)	−0.08 (0.08)
Percentage of population female	8.60*** (1.77)	1.65** (0.48)
Percentage of population non‐Hispanic Black	0.80 (0.56)	0.34* (0.15)
Percentage of population Hispanic	1.77*** (0.47)	0.39*** (0.11)
Percentage of population over age 65	−0.94 (1.08)	−0.29 (0.27)

Notes

Presented are coefficient estimates from a state‐level fixed‐effects regression weighted according to county population. Robust standard errors are in parentheses.

***P < 0.001; **P < 0.01; *P < 0.05.

Stratified regression analyses indicate that the strength of the association between food swamp severity and all‐cause hospitalizations among adults with diabetes differs markedly by rural‐urban context (see Table 3). The association is similar but slightly greater in magnitude in mostly rural counties compared to mostly urban counties; a one point higher food swamp score is associated with an average of 60.42 additional hospitalizations in mostly rural counties (*P* = 0.011, 95 percent CI = 14.04, 106.80) compared to an average of 56.86 additional hospitalizations in mostly urban counties (*P* = 0.005, 95 percent CI = 17.33, 96.39), controlling for all covariates. However, the association is considerably stronger in completely rural counties, in which a one point higher food swamp score is associated with an average of 82.73 additional hospitalizations per 1000 diabetic adults (*P* = 0.022, 95 percent CI = 11.97, 153.49), adjusting for all covariates. For context, a one standard deviation higher food swamp score is associated with an average of 21.32 additional hospitalizations per 1000 diabetic adults in mostly urban counties (*P* = 0.005, 95 percent CI = 6.50, 36.14), 22.66 additional hospitalizations per 1000 diabetic adults in mostly rural counties (*P* = 0.011, 95 percent CI = 5.26, 40.05), but 31.02 additional hospitalizations per 1000 diabetic adults in completely rural counties (*P* = 0.022, 95 percent CI = 4.49, 57.56), controlling for all covariates.

**Table 3:** The Association of Food Swamps on Hospitalization Rates among Diabetic Adults by Rural‐Urban Continuum Category

	All counties (N = 784)	Completely rural (N = 121)	Mostly rural (N = 309)	Mostly urban (N = 354)
*All‐cause hospitalization rate*				
Food swamp score	49.79** (15.54)	82.73* (35.66)	60.42* (23.56)	56.86** (20.09)
*ACSC hospitalization rate*				
Food swamp score	19.12*** (4.09)	15.18 (9.26)	8.87 (6.75)	23.23*** (5.31)

Notes

Presented are coefficient estimates from state‐level fixed‐effects regressions weighted according to county population. Estimates are adjusted for average number of comorbidities per patient, percentage of patients receiving Medicaid, percentage of hospitalizations admitted in ER, percentage of population female, percentage of population non‐Hispanic Black, percentage of population Hispanic, and percentage of population over age 65. Robust standard errors are in parentheses.

***P < 0.001; **P < 0.01; *P < 0.05.

Stratified analyses of ACSC hospitalization rates revealed that a one unit higher food swamp score is associated with an average of 23.22 additional ACSC hospitalizations per 1000 diabetic adults in mostly urban counties (*P* < 0.001, 95 percent CI = 12.79, 33.67). However, no significant association was found between food swamp scores and ACSC hospitalization rates in completely rural or mostly rural counties.

The ancillary models weighted by inverse propensity treatment weights were consistent with the main analyses. Foods swamps are significantly related to diabetic all‐cause hospitalization rates (*β* = 15.34, *P* = 0.042, 95 percent CI = 0.53, 30.16) and ACSC hospitalization rates (*β* = 4.94, *P* = 0.005, 95 percent CI = 1.32, 8.54). Because the difference in mean food swamp scores between food swamp counties and nonfood swamp counties as determined by the dummy variable is less than one point (mean food swamp scores of 3.06 and 2.48, respectively), the magnitudes of these associations are not out of line with those in the main analyses without propensity score weighting. Thus, we retain the unweighted regression analyses are our main analytic approach.

### 3.3 Sensitivity analyses

Our falsification test indicated that food swamp severity is not significantly related to the rate of mood disorder hospitalizations in the full model or in stratified models.

The multivariate model with population‐adjusted RFEI as the predictor and additionally controlling for median income found county RFEI to be significantly associated with all‐cause hospitalization rates and ACSC hospitalization rates.

Food swamp scores remain significantly associated with both all‐cause and ACSC hospitalization rates when additionally controlling for the percentage of homes that are vacant; in fact, the magnitude of the association is stronger in both models. These results are presented in Table 4. The pattern seen in the stratified analysis for all‐cause hospitalizations also remains.

**Table 4:** The Association of Food Swamps on Hospitalization Rates among Diabetic Adults Additionally Controlling for Percentage Vacant Homes (N = 784)

* *	All‐cause hospitalization rate	ACSC hospitalization rate
Food swamp score	56.38** (16.50)	21.23*** (4.30)
Comorbidities per patient	92.60*** (16.50)	12.79** (4.31)
Percentage of patients on Medicaid	−1.26 (0.80)	−0.31 (0.22)
Percentage of hospitalizations admitted in ER	−0.16 (0.35)	−0.09 (0.09)
Percentage of population female	7.87*** (1.85)	1.41** (0.50)
Percentage of population non‐Hispanic Black	0.80 (0.57)	0.34* (0.15)
Percentage of population Hispanic	1.73*** (0.47)	0.38*** (0.11)
Percentage of population over age 65	−0.49 (1.12)	−0.15 (0.28)
Percentage of households that are vacant	−0.72 (0.48)	−0.23 (0.12)

Notes

Presented are coefficient estimates from a state‐level fixed‐effects regression weighted according to county population. Robust standard errors are in parentheses.

***P < 0.001; **P < 0.01; *P < 0.05.

The models utilizing the number of hospitalizations conditional on the number of adults with diabetes rather than the hospitalization rate indicate that higher food swamp scores remain strongly significantly associated with greater all‐cause hospitalizations and ACSC hospitalization rates.

## 4 DISCUSSION

In analyses adjusted for comorbidity burden, Medicaid enrollment, emergency department utilization, population demographics, and state fixed effects, intensified food swamps were associated with increased average hospitalization rates among adults with diabetes. Across all counties, a one unit higher food swamp score is associated with an average estimated additional 49.79 all‐cause hospitalizations for every 1000 diabetic adults and an additional 19.12 ACSC‐specific hospitalizations for every 1000 diabetic adults. These surplus hospitalizations represent a sizable number of complications, considering that there are roughly 30 million adults with diabetes in the United States.^33^

These findings imply that the food environment may play a role in the health outcomes of adults with diabetes, resulting in diabetic residents of food swamps being at a notable disadvantage with regard to complications and hospitalizations in comparison with their counterparts in better food environments. As such, they add support to population‐wide policies that seek to regulate the food environment, such as zoning restrictions for fast food outlets, as they may help reduce this disparity. The findings also suggest that the food environment could perhaps stymy some health system attempts to reduce health care utilization by adults with diabetes, such as the CMS Medicare Diabetes Prevention Program Model. Future efforts to reduce service utilization among diabetics may want to consider the nature of the food environment when designing interventions and include ways to encourage adults with diabetes living in food swamps to less frequently visit the unhealthy outlets that surround them. Some such interventions currently exist, such as Geisinger Health's Fresh Food “Farmacy,” which provides diabetic patients with meal plans and groceries at the hospital site.^34^ Considering the increased service utilization in food swamps, such efforts may in fact be quite cost‐effective.

The finding that a one unit higher food swamp score is associated with a great many more all‐cause hospitalizations (82.73 per every 1000 diabetic adults) in completely rural counties is particularly striking and warrants further consideration. The model of health access by Penchansky and Thomas^35^ has been adapted for the food environment^5^ and suggests that food access is made up of several dimensions. This analysis focuses on availability, which is only one of these dimensions. The others include accessibility, accommodation, affordability, and acceptability, and it is possible that differences in these food environment dimensions between rural and urban contexts, which have been well noted, may moderate the observed association. For instance, rural areas are primarily served by small grocery stores; any supermarkets that do exist are concentrated in regional hubs.^36^, ^37^ These smaller stores often have a limited, less appealing, and more expensive selection of healthy foods than do the supermarkets found in urban areas.^12^, ^13^, ^14^ Thus, although the ratio of fast food outlets to grocers may be similar in urban and rural areas, this lack of acceptable and affordable healthy options may be spurring rural residents, including those with diabetes, to purchase more unhealthy items. In fact, several studies, including some longitudinal studies, have found a positive association between small grocery stores and increased BMI in contrast to either a negative association or no association between supermarket availability and BMI.^6^, ^38^ In addition, the concentration of supermarkets in regional hubs means that rural residents must travel farther distances than their urban counterparts to access healthy foods, which is also made more difficult by the fact that rural areas often lack any public transportation.^13^, ^36^ This may further entice them to purchase more easily accessible unhealthy foods.

The observed stronger association between food swamps and hospitalization rates in rural counties may simply be the result of rural areas' poorer access to primary care, which is likely to be correlated with access to commercial areas and, thus, access to food outlets. Certainly, our data show that rural counties have fewer primary care physicians than urban counties (36.83 physicians per 100 000 residents in completely rural counties, 49.03 physicians per 100 000 residents in mostly rural counties, and 73.88 physicians per 100 000 residents in mostly urban counties). However, when additionally controlling for the ratio of primary care physicians to county residents in the multivariate regression, food swamp scores remain significantly associated with diabetic all‐cause hospitalization rates and ACSC hospitalization rates. In fact, the association becomes larger in magnitude for all‐cause hospitalization rates in both the full sample and the completely rural sample (all‐cause all counties: *β* = 57.82, *P* < 0.001, 95 percent CI = 26.01, 89.03; all‐cause completely rural counties only: *β* = 99.47, *P* = 0.005, 95 percent CI = 30.36, 168.57; and ACSC all counties: *β* = 19.88, *P* < 0.001, 95 percent CI = 11.41, 28.35).

Testing for underlying mechanisms is beyond the scope of this analysis, but the strength of the association between rural food swamps and diabetic all‐cause hospitalization rates observed in this study is particularly concerning because rural areas are disproportionately impacted by diabetes.^39^, ^40^ Our results indicate a statistically significant difference in mean diabetes prevalence between completely rural and urban counties, with rural counties having a 1.11 percentage point higher prevalence than mostly urban counties. These higher rates coupled with the stronger association, if corroborated, may warrant specifically targeting rural areas with interventions and policies to improve the food environment.

That fact that no association was found between food swamp scores and ACSC hospitalization rates in completely rural and mostly rural counties certainly necessitates discussion. It is plausible that rural environments are simply less conducive to hospitalizations for ambulatory care‐sensitive complications. Perhaps longer distances to hospitals and fewer hospital beds in rural areas result in more residents seeking care for these complications in other venues, while urban residents seek care in their comparatively more accessible hospitals (our data do find that urban counties have a higher percentage of hospitalizations admitted through the emergency department than do rural counties). It is also possible that the stratified samples in this analysis are insufficiently powered to detect a statistically significant association. Even when assessing all‐cause hospitalizations, the samples of completely rural counties and mostly rural counties were slightly underpowered with power estimates of only 0.70. The power estimates for ACSC hospitalization rates for the completely rural and mostly rural samples are 0.44 and 0.30, respectively. In essence, it is likely that food swamps still have the hypothesized association with diabetes exacerbations and the development of ambulatory care‐sensitive complications in rural counties, despite it being unobserved in hospital utilization rates.

### 4.1 Limitations

The study findings should be considered in light of some limitations. First, the analyses are cross‐sectional, which inhibits any claims about the causality or temporal ordering of the food swamp‐hospitalization rate relationship. The results from propensity score‐weighted regression analyses suggest that food swamps may exacerbate diabetes and result in higher hospitalization rates, but these conclusions would be better supported with longitudinal data. When appropriate data become available, changes in food swamp severity and hospitalization rates over time should be analyzed to establish the temporal nature of this relationship. Second, the sample size was limited due to the availability of HCUP SID data. As discussed above, statistical power for our completely rural and mostly rural stratified analyses was less than ideal. Certainly, a larger sample of states or a national sample would improve the validity of our stratified analyses as well as their generalizability to the United States as a whole. National analyses should be pursued when these data are available. Third, our outcome and predictor of interest are both ratios, the use of which may result in spurious correlation. We were able to decompose the measure of hospitalization rates and confirm that food swamp scores are still significantly associated with the number of hospitalizations, conditional on the number of adults with diabetes. However, due to the proprietary algorithm used to adjust for population density and average disposable income, we are unable to decompose the food swamp score and include its numerator and denominator as additional covariates in the model. The food swamp score variable as calculated also exhibits little variation, which may lead to imprecision in our estimates. While our focus on food swamps as a measure contributes to the emerging literature on the predictive validity of relative food environment measures,^8^, ^9^, ^10^, ^11^ studies utilizing alternative relative measures that have more variation and components that can be easily separated should be pursued in the future. Finally, analyses were conducted at the county level, as we were limited by our county‐level contextual data. While a more granular geographic unit of analysis may have yielded more precise results, there are also benefits to using the county as the unit of analysis. Zip codes and block groups may capture the food environment near the home, but most residents travel outside of their immediate areas for work and other daily activities and are therefore likely to be impacted by the food environment of neighboring areas. Counties, however, are more likely to capture the majority of their daily routes and, thus, their exposure.^38^ Additionally, land use and zoning regulations often occur at the county level, so estimates for smaller units may be harder to apply to any potential policy change discussions.^11^ Nonetheless, zip code‐level analyses should be pursued when data are available.

## 5 CONCLUSION

Food swamps appear to be linked to a disparity in hospitalization rates among adults with diabetes. In this analysis, we find that the degree to which unhealthy food outlets outnumber healthier options is associated with all‐cause and ambulatory care‐sensitive hospitalizations among diabetic adults, controlling for relevant health system‐related and sociodemographic covariates. Further, the association of food swamp severity with all‐cause hospitalizations is stronger in completely rural counties. Policy makers and health systems may want to consider the nature of the food environment in future efforts to address disparities in diabetes management, reduce preventable hospital utilization among adults with diabetes, and improve population health.
